# Contemporary Educational Interventions for General Practitioners (GPs) in Primary Care Settings in Australia: A Systematic Literature Review

**DOI:** 10.3389/fpubh.2019.00176

**Published:** 2019-06-27

**Authors:** Christina Maresch Bernardes, Isanka Umayangani Ratnasekera, Joo Hyun Kwon, Sivagowri Somasundaram, Geoff Mitchell, Shaouli Shahid, Judith Meiklejohn, James O'Beirne, Patricia Casarolli Valery, Elizabeth Powell

**Affiliations:** ^1^Department of Population Health, QIMR Berghofer Medical Research Institute, Brisbane, QLD, Australia; ^2^School of Medicine, University of Queensland, Brisbane, QLD, Australia; ^3^Centre for Aboriginal Studies, Curtin University, Perth, WA, Australia; ^4^Orange Sky Australia, Brisbane, QLD, Australia; ^5^Sunshine Coast Hospital and Health Service, Birtinya, QLD, Australia; ^6^Princess Alexandra Hospital, Woolloongabba, QLD, Australia

**Keywords:** GPs, educational, intervention, Australia, primary care

## Abstract

**Background:** The primary purpose of educational interventions is to optimize the clinical management of patients. General practitioners (GPs) play a major role in the detection and management of diseases. This systematic literature review will describe the type and outcomes of educational interventions designed for general practitioners (GPs) in the Australian context.

**Methods:** PubMed, CINHAL, and Scopus databases were systematically searched for studies on educational interventions conducted for GPs in Australia during 1st January 2008 to 11th June 2018. Data collected on the methodology of the interventions, GPs satisfaction regarding the educational intervention, changes in knowledge, confidence, skills and clinical behavior of the GPs. We also assessed whether the acquired clinical competencies had an impact on organizational change and on patient health.

**Results:** Thirteen publications were included in this review. The methods with which educational interventions were developed and implemented varied substantially and rigorous evaluation was generally lacking particularly in detailing the outcomes. The reported GP response rate varied between 2 and 96% across studies, depending upon the method of recruitment, the type of intervention and the study setting (rural vs. urban). The most effective recruitment strategy was a combination of initial contact coupled with a visit to GP practices. Nine of the studies reviewed reported improvement in at least one outcome measure: gaining knowledge, improving skills or change in clinical behavior which was translated into clinical practice. In the 3 pre- and post-intervention analysis studies, 90–100% of the participating GPs reported improvement in their knowledge and attitudes.

**Conclusion:** Education interventions for GPs in Australia had low response (recruitment) and retention (GPs that participated in follow-ups) rates, even when financial benefits or CPD points were used as incentives. Higher GP response rates were achieved through multiple recruitment strategies. Multifaceted interventions were more likely to achieve the primary outcome by improving knowledge, skills or changing practice, but the effect was often modest. Inconsistent results were reported in studies involving the use of multiple contact methods within an intervention and conducting online interventions.

## Introduction

General practitioners (GP) play a vital role in the Australian health care system. About 87% of Australians visit their GP at least once a year (1). Recently, Harrison et al. (2), using a random sample of 8,707 patients at encounters with 290 general practitioners, estimated that about half (47.4%) of Australian patients consulting a GP and one-third (32.6%) of the Australian population have multi-morbidity. GPs are increasingly involved in chronic disease management and prevention (3), and are required to make timely and complex decisions for patient care. This in turn demands the attainment of up to date skills and knowledge regarding management of specific diseases, available treatment modalities and pathways to access them (1). GP trainees in Australia receive a combination of formal teaching and supervised practice managed by an accredited GP supervisor (4). Following the completion of training however, the practice of medicine requires regular appraisal and revalidation, and this is achieved through continuous medical education programmes (CME). These programmes are available in a variety of subject areas, formats and platforms. With evolving technology and evidence regarding the effectiveness of various medical education strategies, the format of these CME programmes has changed over the past years with more online and audio-visual content being integrated with conventional formats (1).

A survey conducted in 2012 involving 2,500 GPs, explored their preferences for CME. The study found that 80% of participants indicated that clinical practice was the key motivation for taking part. The preferred learning formats for GPs were: learning in a group rather than on their own (95%), face-to-face lecture-based formats (83%) and interactive discussion (70%). In contrast, the least preferred format was online self-education (55%) (5). GPs have the opportunity to select and follow an educational programme of their preference. It is the clinician's responsibility to update their knowledge, and participation in CME programmes is mandatory for ongoing registration with the Medical Board of Australia (1). While many interventional studies about GP education have been conducted in Australia little is known about the effectiveness of these interventions. Seminal research by Davis et al. (6) investigating CME strategies found that delivery methods such as conferences or activities without reinforcement have little direct impact on improving practice. Over two-decades later, with the introduction of online technology, this systematic review is interested in following up what changes in GP education have occurred in Australia. This systematic literature review aims to respond the following questions: what types of interventions have been used to educate GPs?; what is the response rate of such studies?; and what are the outcome measures used in these studies of education interventions?

The review was restricted to Australia as the research team plans to conduct a RCT with GPs using an education intervention. The educational intervention studies conducted during the past decade will be examined, focus on the recruitment strategies used, response rates and GP satisfaction with the educational intervention. This review will also examine the reported changes in GP knowledge, confidence, skills and clinical behavior, and assess whether the acquired clinical competencies had an impact on organizational change and on patient health outcomes.

## Materials and Methods

### Study Design and Participants

Eligible for this review were peer-reviewed studies (randomized control, quasi-experimental, cohort and qualitative) that described an educational intervention for GPs. The studies had to be conducted in Australia and published in the English language, between 1st of January 2008 to 11st June 2018. This time period was selected to capture the increasing availability of online technology that was integrated into the education system over the last decade (7). Studies that involved GPs and other healthcare professionals were included if the outcomes were reported specifically for GPs. Books, reviews, clinician review notes, case studies, clinical practice guidelines or recommendations, opinion pieces and commentaries were excluded. This review was registered with the Center for Reviews and Dissemination at the University of York (PROSPERO registration number CRD42018089492).

### Systematic Review Protocol

Four researchers (PV, SS, IR, and JK) independently screened the titles of publications against the inclusion and exclusion criteria and selected potentially eligible publications for review. These selected publications were screened by reading their abstracts. Publications that fulfilled the criteria with their title and abstract were then selected for full text review. Any discrepancies in selecting an article were resolved by discussion among the researchers (SS, IR, and JK) and consulting two other researchers involved in the study (PV and CB).

Three researchers (CB, IR, and JK) assessed the methodological quality of eligible articles using a 14-item checklist adapted from the NIH Quality Assessment Tool for Observational Cohort and Cross-Sectional Studies (8). Disagreements were resolved by discussion among the researchers (JK, IR, and CB) and consultation with another independent researcher (PV).

Using a standardized data extraction sheet, four researchers (SS, IR, JK, and CB) summarized each article selected as “Good Quality” for the review, under the categories of: type of study, study duration, response rate, recruitment strategies, type of intervention used, methods of outcome assessment, outcome and conclusion. The quality of the RCTs were not assessed using the “Consolidated Standards of Reporting Trials” CONSORT guidelines, as the scope of this review was to identify strategies and response rates of recruitment, as well as the type outcome measures. Low response rate studies were not excluded to accurately demonstrate the success rate of these interventions.

### Search Strategy and Data Sources

The systematic review followed the Preferred Reporting Items for Systematic Reviews and Meta-Analysis (PRISMA) guidelines. The electronic search included the PubMed, CINAHL and Scopus databases based on inclusion and exclusion criteria defined for the review. The primary search in abstracts included two main terms and their variations (primary care, general pract^*^, family pract^*^, family physician, or community pract^*^, and educat^*^, train^*^, or teach^*^) and Australia. We refined our search yield by removing duplicates using the EndNote X7 software and IBM SPSS version 22.

### Data Analysis

To review and synthesize the findings, a descriptive approach was utilized. Studies were classified according to the type of interventions used to educate GPs: randomized controlled trial, non-randomized intervention, pre- and post-intervention analysis, qualitative study, and mixed methods. The overall response rates of these studies were reported and examined by location; the outcome measures were examined and classified according to their impact in the improvement of knowledge, skills and translation into clinical practice.

## Results

**Figure 1:**
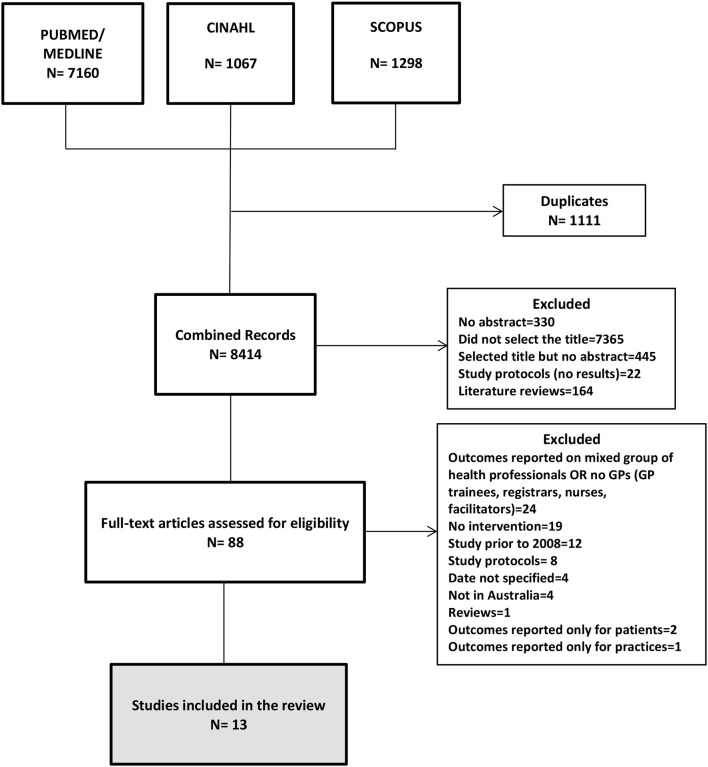
Flowchart of search and selection of articles for review.

The initial search yielded 9,525 results (Figure 1). Following removal of duplicates, there were 8,414 abstracts for assessment of eligibility. Most titles were excluded because the manuscript did not describe an educational intervention for GPs (where GPs were the study's target participants). Eighty-eight articles were selected according to the inclusion and exclusion criteria. After reading the full text, 13 articles were selected for the final systematic review. Eleven were quantitative [six randomized controlled studies (9–14), one non–randomized community intervention (15), three pre- and post-intervention analysis (16–18), one cross-sectional (19)], one was a qualitative study (20), and one used mixed methods (21). The main characteristics of the studies are summarized in Table 1.

**Table 1:** Summary of the study characteristics included in this review.

**Author year, and setting**	**Target disease or GP competencies**	**Study type and duration of intervention**	**Intervention Type**	**Measure**	**Response rate (RR)**	**Outcome**
**RANDOMIZED CONTROL TRIAL**						
Emery et al. (10), Western Australia	Cancer diagnosis	Cluster randomized control trial 2 years	Trial area A—GP education resource card with symptom assessment charts and local cancer referral pathways implemented through multiple academic detailing visits (4 educational practice sessions with case discussions). Trial area B—cancer symptom awareness community campaign tailored for rural Australians (control region)	Total Diagnostic interval (TDI) (defined as the time from first symptom to cancer diagnosis).	69 General practices were invited (RR of GPs was not provided)	Change in practices, timeliness of cancer diagnosis (there was no effect of either intervention)
Paul et al. (12), VIC, NSW, QLD remote towns	Diabetes	Cluster randomized controlled trial 2 years	Online active learning module (ALM approximately 6 h of activities such as quizzes, case studies, self-reflection of activities and moderated peer discussion forum); Direct access to specialist for advice (via online and available on an 'on-call' basis over the duration of the study); and Performance feedback (town based and per GP in their town) provided in the form of a graph (the proportion of patients per GP who were assisted according to the guidelines.	Number of GPs taking up offer of the online learning module; Module completion (website-generated notification) and Access to specialist advice using a log of contact	*N =* 146; (RR = 23%) programme enrolment and 5.5% online module completion and 0% for specialist advice	Change in practices, to improve monitoring and management of diabetes in rural areas through online training;
Harris et al. (11), Four Australian States	Prevention of chronic vascular disease	Cluster randomized controlled trial 1 year	The intervention occurred over 6 months and included: (1) small group training of practice staff (~ 3 h duration using clinical scenarios and case studies to reflect assessment with particular emphasis on lifestyle management of risk factors); (2) feedback on audited performance (from electronic medical record of each practice); (3) a series of practice facilitation visits (based on Quality Improvement (QI): each practice reviewed their performance and set specific goals according to their individual circumstances and resources) and (4) provision of patient education resources and information on referral services and programmes within the local area were provided to each practice in written and electronic form for uploading into practice record systems.	Medical chart audits and survey: Data on the assessment and management of smoking; nutrition; weight; alcohol and physical activity (SNAP); cardiovascular and diabetes risk; blood pressure (BP) and lipids	Eligible Practices *N =* 113 (RR = 24%) GPs *N =* 83 (RR = 84%)	Change in practices, risk recording improved in the intervention but not the control group for weight control, alcohol consumption, smoking status and cardiovascular risk. There was no change in recording of BP, lipids, glucose or BMI and no significant change in the level of risk factors based on audit data. The confidence but not reported practices of GPs and PNs in the intervention group improved in the assessment of some risk factors
Almeida et al. (9), GPs practicing in WA, SA, VIC, NSW, and QLD	Depression and self-harm behavior	Cluster randomized controlled trial	The intervention consisted of: educational material (practical aspects of the assessment and management of depression and self-harm behavior in later life); practice audit of feedback that took place within 6 months of the study, and newsletters outlining progress of the study. Twenty patients attending the practice during the 4 week audit period were included. After each of the 20 consultations reviewed during the audit period, each physician was asked to answer questions (self-completed) about screening mental health problems in the consultation, prescription of anti-depressants, referrals. Physicians assigned in the educational intervention received detailed written audit feedback with number of patients with depression in their practice compared to other participating practices, number of patients with depression and self-harm ideation that they correctly identified compared to control group; the sex, date of birth, specific symptoms of depression reported by each person with depression in their practice audit, and similar information for suicidal ideation.	Measure of the composite of self-harm behavior or clinically significant depression (Patient Health Questionnaire (PHQ-9≥ 10) recorded 12 or 24 months after recruitment (primary outcome)	373 GPs out of 19,046 GPs to whom invitations were sent (RR = 2%)	
Sanci et al. (13), Metropolitan and rural Victoria	GP response to young people's health risks	Randomized control trial 1 intervention, 3 and 12-month follow-ups	Experimental workshops (9 h) in health risk screening, use of a screening tool and motivational interviewing; provision of feedback to clinicians about patients' risk data, and 2 practice visits to support new screening and referral resources	Electronic audit of assessment and recording of health risk behaviors	*N =* 132 practices (RR = 12%) (RR of GPs was not provided)	Change in practices
Shah et al. (14), Metropolitan Sydney	Pediatric asthma	Randomized controlled trial 12 months	The PACE Australia program comprises two structured 3 h small group interactive workshops (up to 10 GPs per group) held 1 week apart. A respiratory pediatrician and community physician lead the topic discussions. A GP presenter discussed the Asthma Cycle of Care which reimburses GPs for two asthma-related consultations within 12 months. The content of the workshops was based on five themes: assessment of the patterns of asthma; appropriate use of medications; provision of Written Asthma Action Plan (WAAP); doctor patient communication and patient education	Proportion of patients who were provided a WAAP; improvement in communication and appropriate prescribing; proportion of children who participated in the Asthma Cycle of Care	Baseline *N =* 150; (RR = 81%) Follow up (RR = 87% of the baseline respondents)	Change in practices
**NON-RANDOMIZED COMMUNITY STUDY**						
Cameron et al. (15), South East QLD, Australia	Management of chronic diseases in adults with severe mental illness	Non-randomized community intervention About 2–3 months	Distribution of guidelines for managing physical comorbidities including the use of brief interventions and motivational interviewing; a website for both health professionals and community members; development of strategies so that clinicians engage and foster ongoing care for people with Severe Mental Illness (SMI) in general practices.	Frequency and length(short vs. long) of GP consultations, type of pathology examinations (obtained via Medicare claims)	(RR = Not clear)	Change in practices. The intervention group had a significant increase in the number and length of consultations for patients with SMI compared to the control group
**PRE- AND POST-INTERVENTION STUDY**						
Morris et al. (18), Australia nationwide	Radiation oncology (RO)	Pre and post intervention analysis study 1.5 years	18 nation-wide sessions conducted between October 2014 and March 2016. Educational sessions within the Radiation Therapy (RT) departments over 2–5 h in the catchment area. The program comprised a RO-led part-didactic, part -interactive presentation, covering basic principles of RT and including 2 clinical cases considered typical of those seen by GPs. Information packs containing relevant materials and information online; Guided tour: included viewing linear accelerators, CT simulator, planning rooms and mold rooms.	Pre and immediate post-session surveys with 12 questions in 4 domains: objective knowledge about RT; understanding referral pathways to RO; self-reported behaviors; feedback on quality and usefulness of educational session. Second survey done 6–8 months later	Baseline Questionnaire *N =* 174; (RR = 96%); Follow-up Questionnaire (6–8 months later) (RR = 38%)	Improved knowledge
Deed et al. (16), Australia wide	Diabetes management	Pre and post intervention analysis study 1 workshop, 3 month follow-up for select groups	Workshops (*N =* 41) focused on multidisciplinary models of care, and reinforcing a simple, best practice process for starting insulin. Reviewed situations that GPs might experience once patients were established on insulin (e.g., inter-current illness, weight gain, management of hypoglycemia and travel). Peer-to-peer group learning was led by diabetes-experienced GP educators. Interactive format utilizing case study approach was used.	Participant learning was reviewed by comparing results of pre-workshop questions on diabetes and insulin management to post-workshop survey	*N =* >2,500 GPs in 41 workshops RR = not provided Post-workshop, *N =* 220 GPs (RR = 22%)	Change in attitudes and practice. GP's confidence in starting and up-titrating insulin improved substantially. Fewer referrals to specialists.
Dev et al. (17), Urban practices in Sydney, Melbourne, Brisbane and Perth	Chronic hepatitis B	Pre and post intervention analysis Phase 1- audit between 2007 and 2008 Phase 2 – ~ 9 months later	Clinical audit structured in a five step format: audit (phase 1) targeting GPs serving communities with high immigrant populations from HBV endemic countries. Each GP identified a minimum of five practice patients that met the inclusion criteria; 3 predefined audit standards were adopted from international guideline recommendations to determine whether GPs were meeting the audit standards. On the completion of phase 1, individual GP results and an educational intervention (no information provided on the sessions) were delivered by nurses. Interval between phase 1 and 2 data collection was approximately 9 months. This allowed GPs to review patient reports from phase 1 and determine whether further tests or referral to a specialist was appropriate. Phase 2 data determined whether GPs were meeting audit standard 1 and 2 only.	Monitoring intervals; counseling and referral	119 GPs in phase 1 and 106 GPs in phase 2; i.e., (RR = 89%)	Change in practice
**CROSS-SECTIONAL STUDY**						
Westaway et al. (19), Australia ‘nationwide	Dementia	Cross-sectional study	GPs received: a mailed pack with educational materials (focusing on addressing environmental, physical and psychosocial factors of patients with dementia and initiating non-pharmacological intervention); the adapted TOP 5 form (for carers to write down practical tips relevant to the care of patients with dementia); and a survey form accessing the acceptability of education materials by GPs	After reading the material, how likely were GPs to assist carers of patients to identify practical tips. Proportion of GPs who received positive feedback from carers regarding helpfulness of sharing their practical tips	*N =* 350 GPs out of 4,827; (RR = 7% survey)	Change in practice
**QUALITATIVE STUDY**						
Boneveski et al. (20), Site not mentioned	“ABC's of vitamin D” program	Internet based, pilot study, audit/ qualitative 2 weeks	Online Continuing Medical Education (CME) program structured in 8 modules. The construct of the program enabled ongoing use, over multiple occasions. Each module contained active learning questions with immediate feedback regarding accuracy of responses. Pre and post-tests were available for those interested in completing the whole program. “Reinforcing activity” involved asking users to review patient education materials	Overall acceptability, module preferences and perceived effectiveness, completion time and qualitative feedback	*N =* 12 (RR = 60%)	Change in practices
**MIXED METHODS STUDY**						
Jones et al. (21), Victoria	Computer assisted chronic disease management	Qualitative study with workshops 8 months	4 learning workshops including plan-do-study-act cycles and a follow-up workshop over a period of 8 months. The program involved a computer assisted chronic diseases management tool and a broadband-based service known as cdmNet. Predisposing activity completed 2 weeks prior to each learning workshop; An evaluation after completion of each module; reinforcing activity completed 2 weeks after each learning workshop	GPs were asked to report on the use of selected Medicare items (e.g., number of General Practice Management Plans (721) that were created for 4 chronic diseases (diabetes, osteoarthritis, cardio-vascular disease and chronic obstructive pulmonary disease) during intervention time	508 invitation letters sent First Workshop *N =* 24 (RR = 5%) Follow up workshop *N =* 15 (RR = 63%)	Change in practices

### GP Characteristics

The total number of study participants varied, ranging from 12 GPs in a qualitative study (20) to a maximum of 373 GPs in a cluster randomized controlled trial (9). The demographic characteristics of GPs were described in 4 studies (11, 13, 14, 20). Two studies (11, 13) reported the distribution of GPs by age ranges with the highest proportion of participants (35–37%) among the age range 45–54 years. A higher proportion (51–61%) of female GPs participated in three studies (11, 13, 14), while the remaining study reported a balanced participation of male and female GPs (20). Two studies reported the proportion of GPs who graduated in Australia [54% (14) and 70% (13)] and the proportion of GPs who graduated before 1989 [54% (13) and 80% (14)]. The working experience of GPs was reported by two studies (11, 14) with the majority of participants [56% (14) and 80% (11)] having <20 years of clinical practice.

### Type of Interventions

Overall, interactive workshops were conducted in six studies (10, 12, 13, 15, 18, 21) educational material including guidelines, screening tools, information sheets, websites were provided in 8 studies (9–11, 13, 15, 17, 18, 20), academic detailing or facilitation visits were conducted in 4 studies (10, 11, 13, 15), feedback of GPs' performance was given in 5 studies (9, 11–13, 20), and online interventions were conducted in three studies (12, 20, 21). The facility to consult experts directly by the GPs had been provided in two studies (11, 12). Among the studies that achieved the expected primary outcome, one (13) had five contact times with GPs, two (11, 21) had 4 contact times, one had 2 (14) and one had a single (15) contact. Two studies (9, 10) with 4 and 3 contact times did not accomplish their primary outcome. Lastly, three studies (11, 13, 21) had nurses and/or other practice staff involved along with the GPs in the educational intervention and achieved their primary outcome with changes in clinical practice.

#### Randomized Controlled Trials

The six RCTs (9–14) reviewed targeted different chronic diseases and GP skill-sets, details are presented in Table 1. Briefly, in four studies (9–11, 13) researchers provided GPs with educational materials (e.g., guidelines, screening tools, and symptom assessment cards), additionally, one study conducted multiple academic detailing visits to educate GPs about the provided materials (10). In three RCTs (11, 13, 14) interventions were based on workshops: in one study a single small group training session based on clinical cases with presentations and role plays using simulated patients (11); another study conducted two small group interactive workshops using presentations and video demonstrations (14); the remaining RCT (13) conducted three interactive workshops with role plays, case discussions and video vignettes, and had provided the GPs with time to reflect on what they encountered during consultation with their patients.

#### Non-randomized Community Intervention

One non-randomized intervention (15) involved a programme to improve the awareness of physical and oral health care needs of individuals with severe mental illness (SMI) in public mental health, primary care, and non-governmental sectors (Table 1).

#### Pre- and Post-intervention Studies

Three pre- and post-intervention studies focused on the management of chronic hepatitis B (17), diabetes (16), and radiation oncology (18) (Table 1). Educational interventions included workshops with interactive peer-to-peer group learning (16) or interactive sessions with case discussions (18) and the distribution of treatment guidelines delivered by nurses (17).

#### Cross-Sectional Study

One cross-sectional study (19) mailed a package containing educational materials about dementia care, an adapted TOP 5 form for carers to write down top practical tips to care for patients with dementia; and a one-page survey response form for GPs to assess the acceptability of the educational materials (Table 1).

#### Qualitative Study

The qualitative study (20) reviewed included an online intervention aimed to improve GP's knowledge and practices regarding vitamin D via an online course with 8 modules. The course content was influenced by results of a baseline survey conducted to assess knowledge deficiencies and weaknesses in managing issues related to vitamin D and sun protection (Table 1).

#### Mixed Methods Study

This study (21) aimed to improve chronic disease management by GPs using four learning workshops, a follow-up workshop and plan-do-study-act cycles over a period of 8 months. Evaluation of the workshops was conducted at three-time points using semi-structured interviews (21) (Table 1).

### Recruitment and Response Rate

#### Overall

The recruitment methods varied across the studies and often combined strategies to improve the response rate (Table 1). Recruitment methods varied from a single one-time invitation letter (9, 10, 16, 19, 21), or gratification points for professional development (CPD) (10, 16) and a combination of invitation letter, followed by a phone call and a visit by a research team member to clarify the study (14). An invitation letter followed by a phone call and a $250 retail voucher was used by another study (20). A study (12) targeting rural GPs utilized six rounds of invitation letters with a total of 10 occasions of contact via primer post card, mailed personalized letter, faxed personalized letter, promotional letter, provision of feedback about the current management of the disease of interest, provision of a brochure promoting online videos for education, and by offering jelly beans, CPD points and $200. One study (10) advertised for expression of interest in research involvement via newsletters, direct mail or phone calls, and an incentive of $1,000 per practice. Three studies did not clearly document their method of recruitment [15, 17, 18].

The response rate for most of the studies was low [varying between 2 and 24% (9, 12, 19, 21)] or was not reported (10, 13, 15–17). Higher response rates [ranging from 60 and 96% (11, 14, 18, 20)], were achieved in studies using a combination of recruitment strategies. The study(18) with the highest response rate (96%) did not state the method of recruitment. The second highest response rate (73.1%) was achieved in a study (14) in which members of the research team contacted GPs who expressed interest in the study and visited the practices. A cross-sectional study (19) that aimed to adapt the a program designed to provide five relevant and meaningful tips to assist people with dementia reported a low response by GPs (350 out of 4,827 GPs) but was highly accepted by participants (90%). GPs indicated they were very likely or moderately likely to assist family members and carers of patients with dementia to identify their top tips to personalize care.

#### By Location

The location of the intervention (rural vs. urban practice) has also been examined. In one study (10, 12) conducted in a rural area, 81% of practices agreed to a single educational session while 58% of practices agreed to participate in the entire programme (4 educational sessions) (10). Another rural study with an online intervention had a GP response rate of only 23% (12). Similarly two studies that were conducted in an urban setting reported response rates of 24% (11) and 73% (14). In one study (13) conducted in both urban and regional practices only 12% of practices agreed to participate in the study. Three studies (9, 18, 19)were conducted nationwide with response rates of 2% (9), 7% (19), and 96% (18). One of these studies (9) had obtained contact details of GPs from the data base of a medical publishing company, while the actual numbers of practicing GPs may be different, this may have led to inaccurate assessment of the response rate. In 4 studies (10, 15, 16, 20) the setting was not stated.

### Outcome Measures

#### Knowledge or Skills Obtained

The knowledge retention or skills obtained by GPs was reported in 3 pre- and post-intervention analysis studies (16–18) in one qualitative study (20) and in one study using mixed methods (21). In the 3 pre- and post-intervention analysis studies (16–18), 90–100% of the participating GPs commented that their knowledge and attitudes improved following the intervention. This change persisted at the 3 month evaluation as increased confidence (71%) and change in behavior (87%) in one study (16). In another study, a 6 month post intervention review (18) reported persistence of increased confidence in 77% of attendees. However, in both studies the post intervention response rate was low, 21.8% (16) at the 3 month assessment and 37% (18) at the 6 months assessment. The remaining study did not assess the long term retention of confidence in the participants (16).

A qualitative study (20)and a study using mixed methods (21) evaluated computer assisted learning with 12 and 15 participants in each. In one study (20) 83% of participants rated the programme as very useful, while the mixed methods study (21) did not describe the proportion of GPs satisfied with the intervention. In the qualitative study (20), 11 of the 12 participants (92%) indicated that they would use the program if it was an accredited CME activity and 75% reported they would refer to the program when talking to patients about related topics. The mixed methods study (21) aimed to promote best practice in GP management of patients with chronic diseases using Medicare Item numbers and broadband-based service (cdmNet). The number of items created and completed initially increased, decreased and then increased again. These results were discussed during the workshops and GPs felt that this pattern occurred because most of the patients requiring a General Practice Management Plan (who were previously not considered for a GPMP) were identified and after that they were managing their “usual” throughput GPs evaluated the learning workshops as being of benefit.

#### Change in Behavior and/or Practice

Following the interventions, researchers expected to see an improvement in knowledge and a change in the behavior or attitudes of participating GPs (evidenced by adherence to guidelines, increased use of a particular software, and increased identification of patients, treatment initiation or specialist referrals).

Of the six RCTs, three studies (11, 13, 14) reported a change in the behavior of GPs at the end of the intervention, while two studies (9, 10) did not observe a difference in behavior. The remaining RCT (12) was withheld because of the low number of GPs recruited, which was inadequate to demonstrate a statistically significant change. The non-randomized control study (15) reported a change in behavior, with the GPs investing more time interviewing adults with chronic mental illness. All three pre- and post-intervention analysis (16–18) studies reported a change in the behavior of GPs, with referral of more patients to specialists (18), treatment initiation in one or more patients post intervention (16, 17) and adherence to guidelines in managing patients (16–18). In the cross-sectional study (19) GPs reported they would be very likely (51%) or moderately likely (38%) to support family members and carers to identify tips that assist in providing care for the person with dementia. The qualitative study did not evaluate a change in practice following the intervention (20). The mixed methods study reported an increase in the use of computer software introduced to the GPs (21).

In the cross-sectional study (19) the acceptability of the intervention by GPs was 90%, and 89% of GPs indicated that they were “very likely” or “moderately likely” to assist family members and carers to identify practical tips to care for dementia patients. In addition, more than a third of GPs (36%) received positive feedback from family and carers regarding the helpfulness of sharing their practical tips to personalize care for their loved ones with dementia. Three RCTs (9, 10, 12) did not achieve the primary outcomes expected.

## Discussion

We reviewed contemporary studies involving GP educational interventions conducted in Australia during the past decade. While this review was restricted to Australia, other countries with similar health system and socio-economic development may face the similar challenges with regards to CME for GPs.

The interventional studies reviewed focused predominantly on improving specific GP competencies by means of improving their knowledge (11, 12, 14, 16, 18, 20, 21) or changing practice (9–11, 13–18, 21). The expected outcome of the interventions was that knowledge would be integrated into medical practice improving the management of specific conditions.

Despite the diverse efforts employed to recruit GPs, through multiple contacts and written materials, most studies had limited success in engaging GPs. Recruitment strategies varied from a single invitation to multiple different approaches including phone calls, financial incentives, CME points and practice visits. A onetime invitation letter was the most frequently used strategy to recruit GPs but resulted in poor response rates compared to other methods. Incorporating multiple recruitment strategies led to a more favorable response rate with the highest rates being recorded when researchers contacted GPs by telephone or by practice visits. Being able to communicate directly with the GPs to clarify their doubts and exchange ideas might have helped to engage GPs, resulting in a higher recruitment.

Offering incentives and CME points did not seem to be an effective strategy in the reviewed studies. This supports the conclusions of Chauhan et al. following an overview of 138 reviews comprising 3,502 individual studies. They found that financial incentives did not have an impact on the long term behavior or practice of family physicians in primary care centers (22).

Continuous professional development is the responsibility of each GP (23). Various circumstances may affect the decision to participate in a study. The driving force is self-motivation, being aware of a knowledge deficit and being driven to restore it. As Taylor and Hamdy describes, the first stage in adult learning, the dissonance phase, is when the learner recognizes a deficiency in his/her knowledge which motivates them to enroll in an educational programme. This challenge to knowledge can be internal; the learner being self-aware, or external, where another person highlights it to the learner (24). The latter strategy was employed in pre- and post-assessment studies where researchers make the participants aware of their level of knowledge prior to the intervention by administering a pre-evaluation. Providing GPs with feedback about their personal performance, lack of adherence to guidelines or impaired recognition of a condition may be a motivating factor for participation in the intervention (24). In a RCT (9) included in the review, researchers provided an audit and feedback incorporating patient responses within the first 6 months of the study, providing GPs with a summary of the prevailing situation. This is further supported by a systematic review conducted by Ivers et al. (25), which revealed that effectiveness of the audit and feedback is greater when the baseline performance is low. They found that the effect of audit and feedback is greater if it is provided by a supervisor or colleague, supplied at multiple instances, delivered in both verbal and written formats and when it includes an explicit target and action plan. Furthermore, according to previous studies, GPs were found to be more willing to participate if the topic or the study content was considered to be relevant and useful in their day to day practice (26).

The format or platform by which the intervention is conducted could be another reason for the choice of participation, retention and gaining expected outcomes in the intervention. Studies included in the review have used multifaceted interventions rather than conducting didactic workshops or distributing printed reading material only. Systematic reviews and meta-analyses show that CMEs conducting multifaceted or mixed interventions that include interactive lectures, case discussions, role plays, video demonstrations, printed educational materials, audits and feed-back are more effective and have a more substantial effect on physicians' performance than interventions involving a single mode of delivery (6, 22, 27–29). A review on behavior change interventions for health care professionals' showed that these interventions can improve knowledge, optimize patients' management, reduce adverse effects and improve patients' outcomes (22). Nine of the 13 studies included in our review used multifaceted interventions. The most common strategies were provision of printed educational materials (8 of 13 studies) and utilizing interactive workshops (6 of 13 studies). These workshops were based largely on clinical cases that GPs encounter in their practice and resulted in positive outcomes (e.g., improved knowledge, increased confidence of GP in prescribing insulin in the primary health setting, GPs investing more time investigating chronic mental issues, increasing referrals to specialists) in most studies.

Slotnick describes that physicians learn by applying the new knowledge to what they have observed and experienced in clinical practice (30), reinforcing the effectiveness of case-based discussions in physician education. Nine of the 13 studies in our review used clinical case discussions as a part of their workshops, academic detailing sessions, practice visits or online educational programmes, to emphasize key points to the GPs. A recent review by Mclean concludes that case-based learning is a method used worldwide in healthcare-related fields to enhance deeper learning in an individual (31).

Educational interventions with longer or multiple contact times were reported to be associated with a better outcome with regards to a behavioral change (28). This was not observed in the studies included in this review.

The meta-analysis conducted by Mansouri et al. notes that interventions targeted at a single discipline group with less participants were associated with better outcomes possibly related to the ability to deliver focused intervention and the opportunity for participants to be actively involved in discussions (28). In the current review, primary outcomes were achieved in all three studies that included nurses/practice staff in addition to GPs.

With advancing technology, internet based learning is recognized as a useful method of providing education, as participants can study remotely, avoiding traveling long distances to participate in workshops. It also provides flexibility, as lack of time or being overcommitted is a key reason given by GPs for withdrawal from a study (32). According to Australian statistics, 86% of households have access to the internet, but the proportion of users accessing the internet for formal educational activities is around 30%, in contrast to entertainment, banking and social networking (all 80%) (33). A study conducted in four GP divisions in Queensland, including two metropolitan, one provincial and one rural division, reported that 89% of GPs have access to the internet in their home or surgery (26). A recent systematic review of online CME programmes conducted in Australia suggests that positive outcomes can be expected in such interventions, particularly with respect to GP satisfaction, knowledge, and practice (34). The three studies included in this review had smaller numbers of GPs with two studies reporting pilot projects (20, 21) and the other study was withheld due to low GP recruitment (12). Therefore, we are unable to draw conclusions about the acceptability of online CME programmes and we believe that further studies are warranted.

## Conclusion

Of the 13 studies we reviewed, favorable response rates were obtained when multiple recruitment strategies were employed. The GP response rate was not influenced by the setting (rural vs. urban), or by offering financial incentives or CME points. Multifaceted interventions resulted in improved knowledge and change in GP behavior. Inconsistent results were reported in studies involving the use of multiple contact methods within an intervention and conducting online interventions. Further study is needed to develop educational interventions that are feasible and able to achieve higher engagement of GPs.

## Author Contributions

CB, PV, and EP: conceptualization. CB, IR, PV, JK, and SiS: data collection. CB, IR, JK, ShS, PV, and EP: formal analysis. CB, IR, PV, and EP: methodology. CB and IR: writing review and editing. CB, IR, JK, SiS, GM, ShS, PV, EP, JM, and JO: writing original draft. All authors have read and approved the final version of the manuscript.

### Conflict of Interest Statement

The authors declare that the research was conducted in the absence of any commercial or financial relationships that could be construed as a potential conflict of interest.
